# Redesigning Nurse–Patient Rounds to Advance Patient-Centered Care in Comprehensive Nursing Service Wards

**DOI:** 10.1093/geroni/igaf122.4182

**Published:** 2025-12-31

**Authors:** Sooyoung Park, Youngmi Lim, Heekyung Kim, Jungha Lee, Soojin Roh, Jiwon Kim, Youngjun Yoo, Dojin Jeon

**Affiliations:** Samsung Medical Center, Gangnam-gu, Seoul, Republic of Korea; Samsung Medical Center, Gangnam-gu, Seoul-t’ukpyolsi, Republic of Korea; Samsung Medical Center, Gangnam-gu, Seoul-t’ukpyolsi, Republic of Korea; Samsung Medical Center, Gangnam-gu, Seoul-t’ukpyolsi, Republic of Korea; Samsung Medical Center, Gangnam-gu, Seoul-t’ukpyolsi, Republic of Korea; Samsung Medical Center, Gangnam-gu, Seoul-t’ukpyolsi, Republic of Korea; Samsung Medical Center, Gangnam-gu, Seoul-t’ukpyolsi, Republic of Korea; Samsung Medical Center, Gangnam-gu, Seoul-t’ukpyolsi, Republic of Korea

## Abstract

Comprehensive Nursing Service wards in Korea provide 24-hour clinical and daily care by nurses and nurse assistants without family presence, aiming to reduce caregiver burden and ensure equitable care. However, among older patients—many postoperative with high treatment needs—patient experience surveys revealed gaps, including difficulty voicing concerns, limited participation in decisions, and insufficient emotional support. Although a “Happy Rounding” program—routine nurse–patient rounds to proactively identify needs—was in place, nurse surveys indicated inconsistent practice due to lack of standardized criteria, varied interpretations of rounding procedures, and uncertainty about its role in care. This study aimed to develop and evaluate a ward-specific Happy Rounding protocol to enhance proactive nursing and improve patient experience. Through team discussions among nurses, a ward-specific protocol was co-designed into the “Double Happy Rounding Checklist.” To enhance nurse engagement, we introduced ward-wide visual reminders and a patient experience process including bedtime pain checks, clear explanations of procedures and medications, and consistent use of empathic language and active listening. A two-week pilot test with patients and nurses confirmed feasibility, guided refinements, and led to ward-wide adoption. Implementation improved nurse involvement and patient satisfaction. Patients reported that nurses listened more attentively (89.9→96.5), responded more promptly (88.9→97.2), explained procedures more clearly (87.0→97.9), and created a safer environment for raising concerns (80.1→94.3). In addition, 81.2% of nurses endorsed the necessity of Happy Rounding, reflecting strong acceptance. This innovation demonstrates that proactive, standardized rounding can strengthen trust, safeguard patient rights, and advance patient-centered care, with future work needed to sustain and scale its impact.

